# Autologous circulating angiogenic cells treated with osteopontin and delivered via a collagen scaffold enhance wound healing in the alloxan-induced diabetic rabbit ear ulcer model

**DOI:** 10.1186/scrt388

**Published:** 2013-12-30

**Authors:** Aonghus O’Loughlin, Mangesh Kulkarni, Erin E Vaughan, Michael Creane, Aaron Liew, Peter Dockery, Abhay Pandit, Timothy O’Brien

**Affiliations:** 1 Regenerative Medicine Institute, National University of Ireland, Galway, Ireland; 2 Network of Excellence for Functional Biomaterials, National University of Ireland, Galway, Ireland; 3 Department of Anatomy, National University of Ireland, Galway, Ireland

## Abstract

**Introduction:**

Diabetic foot ulceration is the leading cause of amputation in people with diabetes mellitus. Peripheral vascular disease is present in the majority of patients with diabetic foot ulcers. Despite standard treatments there exists a high amputation rate. Circulating angiogenic cells previously known as early endothelial progenitor cells are derived from peripheral blood and support angiogenesis and vasculogenesis, providing a potential topical treatment for non-healing diabetic foot ulcers.

**Methods:**

A scaffold fabricated from Type 1 collagen facilitates topical cell delivery to a diabetic wound. Osteopontin is a matricellular protein involved in wound healing and increases the angiogenic potential of circulating angiogenic cells. A collagen scaffold seeded with circulating angiogenic cells was developed. Subsequently the effect of autologous circulating angiogenic cells that were seeded in a collagen scaffold and topically delivered to a hyperglycemic cutaneous wound was assessed. The alloxan-induced diabetic rabbit ear ulcer model was used to determine healing in response to the following treatments: collagen seeded with autologous circulating angiogenic cells exposed to osteopontin, collagen seeded with autologous circulating angiogenic cells, collagen alone and untreated wound. Stereology was used to assess angiogenesis in wounds.

**Results:**

The cells exposed to osteopontin and seeded on collagen increased percentage wound closure as compared to other groups. Increased angiogenesis was observed with the treatment of collagen and collagen seeded with circulating angiogenic cells.

**Conclusions:**

These results demonstrate that topical treatment of full thickness cutaneous ulcers with autologous circulating angiogenic cells increases wound healing. Cells exposed to the matricellular protein osteopontin result in superior wound healing. The wound healing benefit is associated with a more efficient vascular network. This topical therapy provides a potential novel therapy for the treatment of non-healing diabetic foot ulcers in humans.

## Introduction

Diabetic foot ulceration is the most common reason for hospitalization in people suffering from diabetes mellitus [1]. Despite conventional treatments, there exists a high amputation rate. Diabetes-related lower extremity amputations arise from pre-existing ulceration in approximately 85% of cases [2]. Topical cell-based therapy offers a potential new treatment for non-healing ulcers and may prevent the need for amputation. Normal cutaneous wound healing is a complex biological response to trauma, involving the sequential activation and integration of several biological processes [3-5]. These processes include coagulation, inflammation, chemotaxis, angiogenesis and tissue remodelling. There are interactions of many different cell types and cytokines to allow normal wound healing. Delayed wound healing as occurs with diabetes mellitus results from dysregulation of this process [6-9].

Endothelial progenitor cells (EPCs) are a recently identified cell type which promote neoangiogenesis (new blood vessel formation arising from pre-existing blood vessels) and neovasculogenesis (*de novo* blood vessel formation) [10]. Circulating angiogenic cells (CACs) have previously been described as early EPCs and are easily isolated from the mononuclear cell fraction of peripheral blood [11]. More specifically, CACs are low density mononuclear cells from peripheral blood that are plated on fibronectin in media supplemented with endothelial growth factors and fetal calf serum. These adherent cells promote neovascularization predominantly by paracrine effect. CACs are defined by culture methods and staining of acetylated low-density lipoprotein and lectin. They are isolated and cultured in the same manner as early EPCs [12-14].

CACs have been shown to be involved in wound healing and are recruited to sites of neovascularization in the granulation tissue, where they help release various cytokines that facilitate wound repair [14]. In the diabetic state, CACs are reduced in number and function and contribute to the poor wound healing response seen in diabetic ulceration [15]. CACs are constantly in the circulation and cutaneous wounding leads to increased homing of CACs to the wound. This arises from ischemia induced upregulation of stromal cell-derived factor-1α. In addition CACs are released from bone marrow in response to wounding. This process is impaired with diabetes [6]. Transplanting CACs into the wound has been reported to increase recruitment of macrophages and promote revascularization, resulting in accelerated healing [16].

CACs are reduced in number and are dysfunctional in those with poorly controlled diabetes as compared to well controlled diabetes. There are a variety of factors which lead to differing levels of CACs. These include, but are not limited to, smoking, diabetes, hypertension and statin medication. CACs isolated from people with diabetes demonstrate reduced angiogenic potential as demonstrated by reduced tubule formation in the matrigel assay. Diabetic CACs demonstrate reduced adhesion to matrix proteins and reduced migration [17,18]. Our group has recently observed decreased expression of the matricellular protein osteopontin (OPN) in CACs from patients with type 1 diabetes mellitus in the absence of microvascular or macrovascular complications. OPN is a secreted glycoprotein that is involved in cell migration, cell survival, regulation of immune cell function, inhibition of calcification, control of tumor cell phenotype and has been implicated in the process of neovascularization in various cancer models and peripheral ischemia [18].

CAC dysfunction has been reported to be reversed by exposure to OPN. The exposure of CACs from OPN knockout mice to recombinant OPN augments angiogenesis in a mouse hind limb ischemia model [18]. Autologous CACs can be manipulated *ex vivo* to augment cellular function. After isolation and culture of CACs, the cells which are in culture can be manipulated to augment the angiogenic potential of CACs. This provides an attractive autologous topical therapy with CACs exposed to OPN for the treatment of diabetic ulceration.

Conventional cell transplantation techniques using systemic intravenous injection or local intradermal injection results in low cell survival. Cells when injected intravenously typically do not track to the wound site. They are found in the lungs, spleen and liver [19,20]. There exists a reduced blood supply to diabetic ulcers due to peripheral vascular disease, potentially reducing the number of transplanted cells reaching wounds from the systemic circulation. A collagen scaffold is an effective biomaterial to topically deliver cells to a wound and allows for sustained viability of cells in addition to maintaining cells at the wound site. Collagen is a constituent of the extra-cellular matrix and has been established for tissue engineering and cell therapy. It provides support for cell growth and attachment.

The hypothesis tested in this study was that topical administration of autologous CACs applied to a hyperglycemic full thickness cutaneous ulcer enhances wound healing. In addition to this, the pre-activation of CACs by OPN can further enhance the wound healing process.

## Methods

### Collagen extraction, scaffold formation and cell seeding

**Figure 1:**
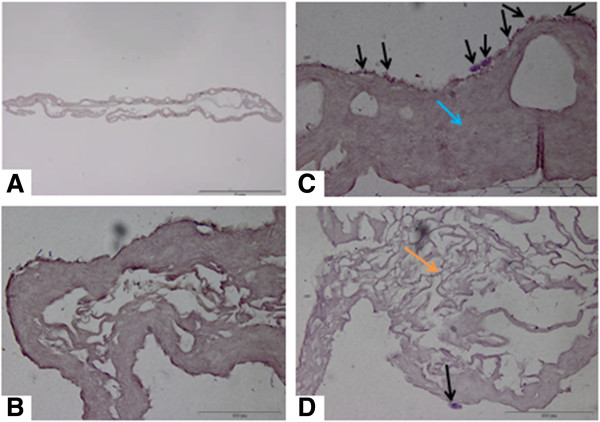
**H & E section of rabbit CACs seeded in a collagen scaffold.** Image **A** is a view of the cell seeded scaffold (Scale bar = 1 mm). Image **B** is a control scaffold without any CACs seeded (Scale bar = 100 μm). Image **C** demonstrates the cell seeded scaffold treatment pre-transplantation. The black arrows demonstrate CACs having been seeded on the scaffold for 24 hours. CACs are blue cells, and are predominantly located on the surface of the scaffold. The blue arrow points to the collagen scaffold which is homogeneous in appearance. There are large pores visible in this area of the scaffold. (Scale bar = 100 μm). The surface of the scaffold to which the cells attach is applied directly to the wound bed ensuring the attachment of cells in that area. Image **D** demonstrates another area of the scaffold which is more porous collagen. This porous appearance of the scaffold was observed at the periphery of the scaffold (orange arrow). A CAC attached to the scaffold is depicted by the black arrow (Scale bar = 100 μm). CACs, circulating angiogenic cells.

Type 1 bovine collagen solution was isolated and purified as described previously [21]. A collagen sponge was fabricated by pipetting 1 mL of 3% (weight) type 1 atelocollagen solution into 24 well tissue culture plates (Sarstedt Ltd, Numbrecht-Rommelsdorf, Germany). This solution was then lyophilized overnight using a VirTis freeze-dryer (Ipswich, Suffolk, UK). The collagen sponge was then washed with Hanks balanced salt solution (Sigma-Aldrich, Arklow, Wicklow, Ireland), three washes with 70% ethanol, two washes of sterile water (Sigma-Aldrich, Arklow, Wicklow, Ireland), and two washes of supplemented EBM-2 (Lonza Swords, Ireland) media. After the washing steps the collagen scaffold was transferred to one well of a 48 well cell culture plate (Sarstedt). This was to ensure the scaffold was occupying the base of the well. Cells were seeded by injecting 1 × 10^6^ in 500 μl of EBM-2 using an insulin syringe (Becton, Dickinson and Company, Oxford, UK) and placed in an incubator for 24 hours at 37°C and 5% CO_2_. The optimum time required for cell seeding on the scaffold was assessed by measuring metabolic activity of cells at 6, 12 and 24 hours after seeding. Figure 1 presents a representative image of the cell seeded collagen scaffold pre-implantation.

### Assessment of metabolic activity and cell viability

The metabolic activity of cells was assessed using the alamarBlue® (resazurin, Invitrogen, Carlsbad, CA, USA) assay. This assay assesses oxidation and reduction reactions in mitochondria. When added to cell cultures, the oxidized form of alamarBlue® enters the cytosol and is converted to the reduced form by mitochondrial enzyme activity by accepting electrons from NADPH, FADH, FMNH, NADH as well as from the cytochromes. NADH is required for oxidative phosphorylation and ATP generation. This redox reaction is accompanied by a change in color of the culture medium from blue to florescent pink, which can be measured by colorimetric or fluorometric means. Experiments were performed in triplicate. CACs were washed once with Hanks balanced salt solution and incubated in alamarBlue® for 24 hours. The percentage reduction in the dye was assessed as above. This was repeated at 24, 48 and 72 hours. The absorbances of each sample were measured in a 96-well plate at wavelengths of 550 and 595 nm using a microplate reader. The percentage of reduced alamarBlue® was determined as previously described [22].

### *In-vivo* model

Eleven male New Zealand white rabbits (3 to 3.5 kg) were used in the study. The protocol was approved by the ethics committee of the National University of Ireland, Galway and the study conducted under a license granted by the Department of Health and Children, Dublin, Ireland. Rabbits were housed in individual cages and with a 12-hour light/dark cycle and controlled temperature and humidity. Rabbits were fed a standard chow and water *ad libitum*.

### Induction of hyperglycemia

Nine rabbits were sedated with intramuscular injection of ketamine, xylazine and acepromazine. Hair was shaved off the back of the ears. Alloxan (150 mg/kg) (Sigma-Aldrich) was made up in 30 mL of saline and administered via an ear vein using an intravenous cannula at a rate of 1.5 mL/minute. After treatment, water containing glucose was provided for 24 hours to prevent hypoglycemia in addition to provision of molasses to the animals’ front paws to avoid hypoglycemia. Blood glucose was checked daily from the marginal ear vein for the first week using Accucheck® advantage strips (Roche, Dublin, Ireland). Blood sugar levels were then checked weekly after the blood sugar level had stabilized. Insulin therapy was administered if the animal lost weight and had ‘high’ glucose readings on a glucometer (indicating blood glucose greater than 33 mmol/L), using insulin glargine (Sanofi-Aventis, Paris, France). Two rabbits were not treated with alloxan and were considered non-diabetic controls.

### Autologous circulating angiogenic cell isolation and culture used for *in vivo* studies

Four weeks post-alloxan treatments, rabbits were anesthetized using intramuscular acepromazine (0.1 mg/kg) and inhaled isofluorane anaesthesia. A total of 10 mL/kg of blood was withdrawn from the marginal ear artery and collected in lithium heparin coated blood collection tubes (BD Biosciences, Oxford, UK). CACs were then isolated as previously described [23]. Briefly, the blood was mixed 1:1 with Hanks balanced salt solution (Sigma) and layered on Ficoll-Paque™ PLUS (GE Healthcare, Cork, Ireland). The sample was centrifuged and the buffy coat was removed to isolate the peripheral blood mononuclear cells. The cells were then washed with red cell lysis buffer (Sigma) and subsequently with phosphate buffered saline (PBS) and re-suspended in EBM-2 media (Lonza). The media was complete media and supplemented with 2% fetal bovine serum, hydrocortisone, fibroblast growth factor, insulin-like growth factor-1, ascorbic acid, epidermal growth factor and GA-1000. Cells were plated on two wells of a fibronectin coated six-well plate (BD Biosciences). After four days EBM-2 medium was changed and recombinant OPN (Sigma-Aldrich) was added to half the cells at a concentration of 5 μg/mL for 24 hours. The medium was changed with no OPN added to the other half of CACs. Cells were trypsinized with 0.25X trypsin/ethylenediaminetetraacetic acid (EDTA) (Sigma). Cell viability was assessed by trypan blue exclusion using a hemocytometer. A total of 1 × 10^5^ CACs was the maximum number of autologous CACs which were able to be isolated from each animal. A total of 5 × 10^4^ CACs and 5 × 10^4^ CACs exposed to OPN were seeded in a collagen scaffold for 24 hours as described above. The upper surface of the CAC-seeded scaffold was applied to the base of the ulcers.

### Surgical procedure

**Figure 2:**
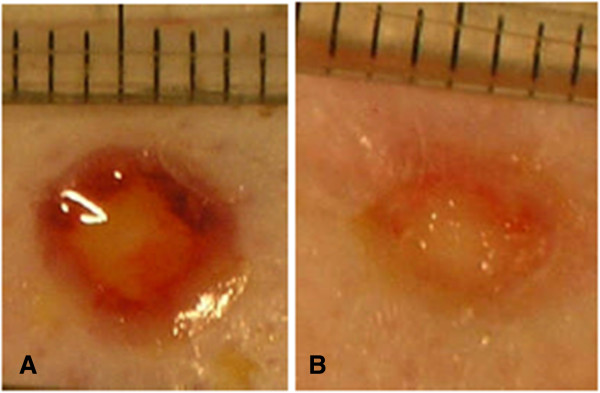
**Gross images of untreated wounds. A)** Representative gross picture of diabetic wound after one week. **B)** Representative gross picture of non-diabetic wound after one week. (Scalebar = millimetres).

Six days after phlebotomy, rabbits were anesthetized using intramuscular injection of 0.1 mL/kg xylazine and 0.12 mL/kg ketamine which is half dose analgesia. Sterile disposable 6 mm punch biopsies (Panvet, Ireland) were used to create two wounds on each ear. A total of four full-thickness punch biopsy wounds were created on each of the nine hyperglycemic animals. The wounds were created and the dermis exposed to bare cartilage. Each wound was treated with one of the four randomized treatment groups: untreated wounds, collagen scaffold alone, collagen scaffold seeded with 5 × 10^4^ CACs, collagen scaffold seeded with 5 × 10^4^ CACs exposed to OPN. The wounds were covered with a polyurethane dressing (Opsite®, Smith & Nephew, Dublin, Ireland) and the ear was sutured and covered with adhesive dressing, (Operfix™, Promedicare, Dublin, Ireland) until day seven (n = 8). On day seven, rabbits were euthanized with intravenous sodium pentobarbital (2 mL). Twenty full-thickness punch biopsy wounds were created in two non-diabetic animals. Five full-thickness punch biopsy wounds were created on each ear of the two non-diabetic animals. Figure 2 demonstrates representative images of diabetic and non-diabetic wounds. Wounds were covered using an adhesive dressing (Opsite®). One hyperglycemic animal was used to detect fluorescently labeled CACs in the wound. This animal was sacrificed at one week.

### Wound closure

At necropsy, ears were surgically removed and the wound area was traced. Wound closure was assessed using Formula A. The area of the wound was determined by measuring the pixels within the tracing and was assessed by Cell B software™ (Olympus). Wound closure was assessed in both the diabetic and non-diabetic ulcers.

$$\%C=\frac{A_{0}-A_{i}}{A_{0}}\times \frac{100}{1}$$

Formula A: Percentage Wound Closure (%C): A_0_ is the area of the wound at day 0 and A_i_ is the area of the wound at day seven as measured by wound tracings.

### Histology

The explants were cut across the midline and fixed in 10% formalin for 24 hours. The tissue was processed and embedded in paraffin. Sections (5 μm) were taken when the tissue was reached. Nine sections were cut using a microtome every 150 μm into the wound for analysis. Three sections were placed on one slide. Sections were stained with hematoxylin and eosin using standard protocols.

### Cell labelling

CM-DiI (Invitrogen) was used to fluorescently label cells for one animal experiment. Briefly, CACs were incubated with CM-DiI at a concentration of 4 μM for 20 minutes. The histological sections were obtained as above and the cells visualized using a fluorescence microscope in the TRITC-rhodamine channel.

### Wound volume

**Figure 3:**
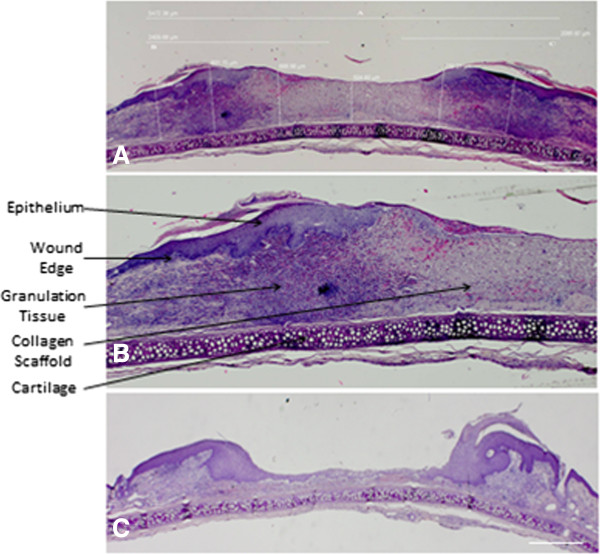
**H & E section one week post treatment. A)** demonstrates an example of a wound one week after CAC + collagen application. Wound thickness was calculated by measuring six arbitrary wound thickness lengths taken from the cartilage to the wound surface and the average thickness obtained. Percentage epithelialization is also measured (A,B,C) Scale bar = 1 millimeter. **B)** demonstrates one half of the wound at increased magnification to further define the tissue architecture. The wound edge is defined by a change in thickness of the epithelium, lack of normal skin appendages and disorganized collagenous tissue. The collagen scaffold and new granulation tissue formation is highlighted. This is the area where neoangiogenesis and neovascularization occurs and where further stereological analysis was undertaken. **C)** is an example of an untreated wound. There is decreased wound healing as evident by a wound with little epidermal tissue formation. There is less granulation tissue, angiogenesis and collagen observed in the control wound. This is likely due to the nature of the wound creation which is a full thickness wound created with a 6 mm diameter punch biopsy. The wound heals from the edges and this is the reason for the morphology seen in untreated wounds.

Images were obtained at 2× magnification using an inverted microscope (Olympus BX51). The thickness of the wound was measured from the top of the wound to the cartilage (Figure 3A). This was performed at six arbitrary points across the wound and an average of six readings calculated. Cell B software was used for analysis (Olympus). For each wound the average thickness of the wound was multiplied by the area obtained from the tracing of the wound at day of necropsy to calculate wound volume.

### Epithelialization

Epithelialization was assessed by measuring the horizontal distance between the two wound edges (Figure 3A (A)). The wound edge was determined by a change in thickness of the epithelium, a lack of sebaceous glands, hair follicles and skin appendages. The length of the newly formed epithelium was measured (Figure 3A (B + C)) and a percentage was obtained. This was performed on the first section analyzed from the center of the wound.

### Stereology

Stereology is a means of assessing tissue responses to tissue constructs [24]. It allows assessment of angiogenesis in vascular beds [25]. In the present study vertical sections of the tissue were examined using a systematic random sampling strategy to provide estimates of relevant stereological parameters [26].

**Figure 4:**
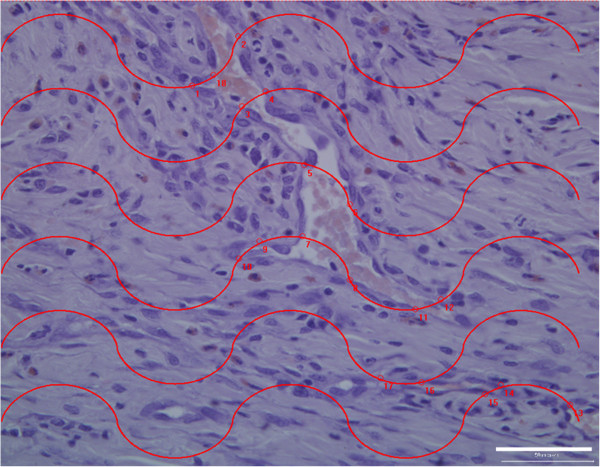
**Enumerating blood vessels using stereology.** A representative image of a hematoxylin and eosin stained histological section at 20X magnification is shown. A series of cycloid lines are electronically placed on the section. The points where the grid intersected blood vessels are numbered on the section. Similar to Figure 3B, the part of the wound analyzed is the area where neovascularization occurs. Scalebar = 50 μm.

**Figure 5:**
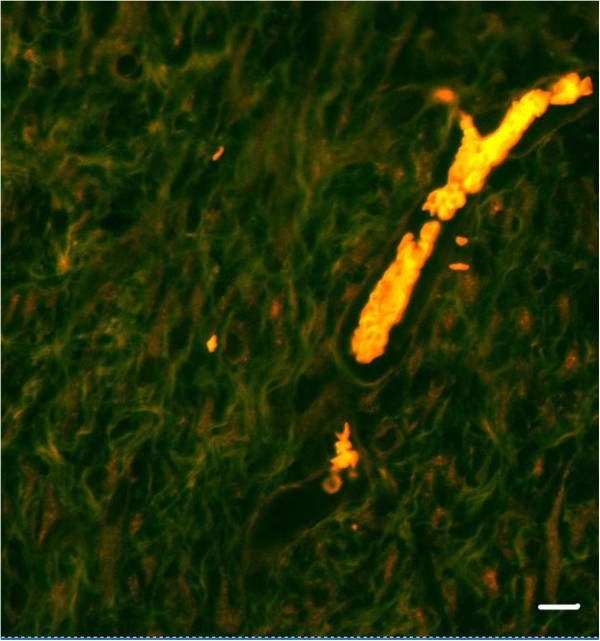
**Identifying blood vessels using fluorescence microscopy.** A representative image of a blood vessel in a H & E stained histological section is demonstrated. The blood vessel appears as a red structure under fluorescence microscopy. This technique was used to identify blood vessels in a H & E stained histological tissue if there was a question over tissue distortion or artifacts in a histological section appearing as a blood vessel. This increases the accuracy of enumerating blood vessels in histological sections. Scalebar = 30 μm.

Methods used to measure the length of vessels in three dimensions were based on the work of Gokhale using a vertical orientation design and a cycloid test system [27]. A series of cycloid lines were placed on the histology sections using Image Pro® Plus software (Figure 4). A systematic random sampling strategy was employed throughout this study. Five fields of view were obtained across the wound bed from one edge of the wound to the other edge [28]. The fields were captured at 20X magnification. To ensure blood vessels were not artefacts in tissue, fluorescence microscopy was used to successfully identify vascular structures. Figure 5 demonstrates blood vessel identification using fluorescence microscopy [25].

The parameters assessed were surface density of blood vessels, length density of blood vessels and radial diffusion distance between capillaries [24,28]. The surface density of blood vessels was calculated using Formula B and the length of the test line was 2,400 nm. The surface area of blood vessels was then calculated by multiplying the surface density by wound volume. To calculate the length density of blood vessels, a series of cycloid lines measuring 2,240 nm in length was rotated 90 degrees and placed on the histological section. The length density of blood vessels was calculated using Formula C. The total length of blood vessels in the wound was calculated by multiplying length density by wound volume. The radial diffusion distance was calculated using Formula D. The radial diffusion distance allows for the measurement of the distance between blood vessels and is an indicator of the efficiency of a capillary network. The smaller the distance between blood vessels, the shorter the distance required for nutrients to diffuse into surrounding tissues. Blood vessel diameter was calculated using Formula E [29]. The blood vessel diameter has been adjusted by multiplying by a shrinkage factor (1.6) that occurs with tissue processing.

$$S_{v}=2\times \frac{I}{L_{T}}$$

Formula B: Surface Density (S_V_), I = number of intersections with test line.

L_T_ = Length of test line 

(2,400 microns)

$$L_{v}=\frac{2\;\times \;\mathrm{IL}}{T_{s}}$$

Formula C: Length Density (L_V_), IL = number of intersections with test line.

T_S_ = thickness of the histological section (5 μm)

$$R_{\mathrm{diff}}=\frac{1}{\sqrt{\pi \;\times \;\mathrm{Lv}}}$$

Formula D: Radial Diffusion Distance (R_diff_), L_V_ = length density.

$$d=\frac{\mathrm{Sv}}{\mathrm{Lv}\cdot \pi }$$

Formula E: Blood Vessel Diameter (*d*) S_V_ = surface density, L_V_ = length density.

### Volume fractions of cells

**Figure 6:**
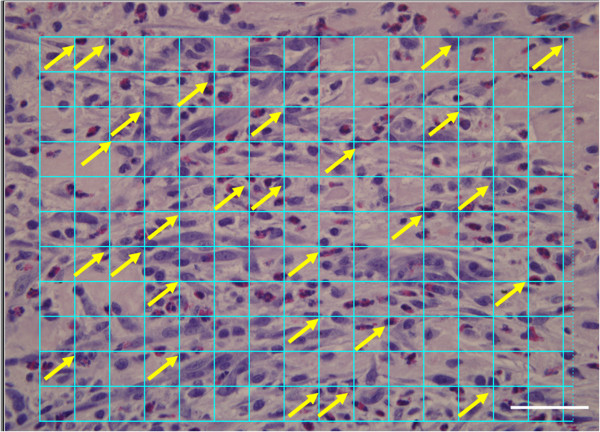
**Calculating the volume fraction of inflammatory cells using a 192 point grid.** A representative image of a hematoxylin and eosin stained histological section demonstrates healing granulation tissue. This area is where neovascularization occurs. A grid is electronically generated and placed on the section. Arrows indicate examples of inflammatory cells on intersection of the grid. Scalebar = 50 μm.

The volume fraction of a feature within a particular reference space can be described as the proportion of space that the feature occupies in a unit volume [28]. Inflammatory cells were counted and included lymphocytes and neutrophils. The volume fraction was determined with a 192 point grid using Image Pro® Plus software (Media Cybernetics) (Figure 6). Neutrophils were identified as small dense circular multi-lobed cells and lymphocytes as small round dense cells with large nuclei. The volume fraction V_v_ is calculated as follows:

$$=\frac{p_{p}}{P_{T}}$$

Formula F: Volume fraction: P_p_ is the number of intersections of the grid on the cell and the P_T_ is the number of intersection on tissue. The volume fraction was multiplied by wound volume.

### Statistical analysis

All bar charts represent mean ± standard deviation. A two sample t-test was used to assess the difference between healthy and diabetic wounds. A one way analysis of variance (ANOVA) was used to assess for a difference across groups at seven days post treatment. Differences between individual groups were assessed using *post hoc* analysis with Fisher’s test and results are reported as 95% confidence intervals. Pearson’s correlation was carried out for each group between the following parameters: percentage wound closure, surface area of blood vessels, length of blood vessels and blood vessel diameter. Minitab® software was used to perform statistics.

## Results

### *In-vivo* model

Animals became hyperglycemic within 48 hours and remained hyperglycemic for the duration of the study. Insulin therapy was required for two animals. Five animals lost weight after alloxan administration ranging from 0.1 to 0.5 kg. Two animals died due to hyperglycemia and are not included in the study.

### Wound closure in diabetic and non-diabetic animals

Wound closure is calculated as in Formula A. As Figure 2 demonstrates, diabetic animals had significantly reduced percentage wound closure at one-week (35.4% ± 9.1) as compared to non-diabetic animals (54.6% ±13.6, Mean ± standard deviation, *P* <0.05, analyzed by two sample t test). This wound healing endpoint is a highly relevant measure as it is non-invasive and readily translatable to the clinic.

### Cell labeling and metabolic activity of rabbit CACs

Figure 1 highlights a representative image of the cell-seeded collagen scaffold. The metabolic activity of the cell-scaffold treatment was assessed. A total of 5 × 10^4^ rabbit CACs demonstrated metabolic activity 24 hours after seeding in a collagen scaffold. This reduced to the same level as the collagen control at 72 hours. No statistical difference in cell viability of CACs exposed to OPN and seeded on a collagen scaffold, and CACs not exposed to OPN seeded on a collagen scaffold was observed. The cells were located at the wound edge and in proximity to cartilage.

### Percentage wound closure in treatment groups

**Figure 7:**
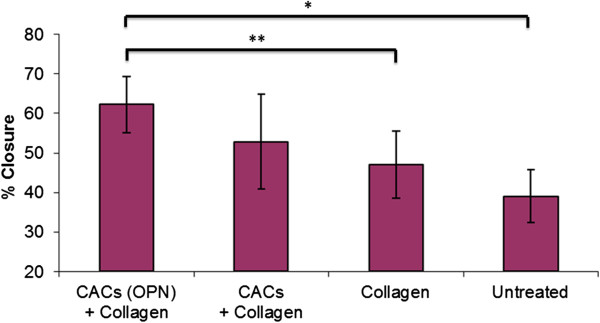
**Percentage wound closures as assessed using wound tracing.** To determine if there was a difference in percentage wound closure, ANOVA was used. For the ANOVA analysis there was a significant difference between groups (*P* = 0.003). The error bars represent standard deviation. The number of wounds per group was eight (n = 8). To determine if there was a difference between individual groups, *post hoc* testing with Fisher’s test was used. The statistical results are reported as 95% confidence intervals (CI). * denotes a significant difference in wound closure ((95% CI (14.109, 32.242)). ** denotes a significant difference in wound closure ((95% CI (6.188, 24.321)). A significant difference is also present when comparing wound closure between CAC + collagen treated wounds and untreated wounds ((95% CI (4.743, 22.877)).

**Figure 8:**
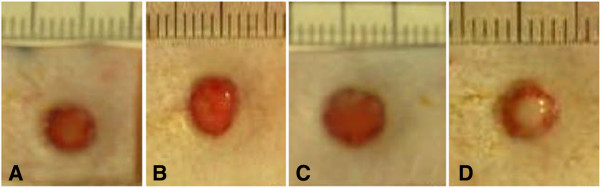
**Representative gross images of wounds.** One week after treatment application, CAC (OPN) + collagen treatment reveals a smaller wound **(A)** when compared to collagen alone **(C)** and untreated wounds **(D)**. Representative images of wounds treated with CAC + collagen treatment (that is, not exposed to OPN) **(B)** appear smaller than wounds treated with collagen alone **(C)** and untreated wounds **(D)**. However, there was no significant difference in percentage wound closure between wounds treated with CACs + collagen **(B)** and wounds treated with collagen alone **(C)**. Scale bar = 1 mm.

The treatment group with CACs exposed to OPN and delivered using a collagen scaffold demonstrated increased percentage wound closure at one week post-treatment as compared to collagen alone and untreated wounds (Figure 7). CACs not exposed to OPN when seeded on a collagen scaffold demonstrated significantly increased percentage wound closure when compared to untreated wounds. There was no significant difference between wounds treated with collagen seeded with CACs and collagen alone. CACs exposed to OPN and seeded on a collagen scaffold did not show a significant difference in percentage wound closure when compared to CACs seeded on a collagen scaffold. Representative images of the wounds in the four groups are presented in Figure 8.

Figure 3 demonstrates representative hematoxylin and eosin stained wounds and epithelialization of ulcers. There was no significant difference in percentage epithelialization between groups. Percentage epithelialization was assessed one week after wound creation and treatment application. The four groups analyzed included untreated wounds or wounds treated with three other treatments.

### Wound volume, stereology and inflammation

At one-week post treatment, collagen alone has a higher wound volume, as compared to CACs exposed to OPN and seeded in a collagen scaffold and untreated wounds. This increased wound volume seen with collagen results in an increased length of blood vessels in the collagen-treated wound and increased surface area of blood vessels in the collagen-treated wound (Table 1). Figure 4 demonstrates the use of a cycloid grid on a histological section for stereological analysis. A significantly increased surface density and length density, in addition to a reduced radial diffusion distance, is noted with all treatment groups in comparison to untreated wounds. Blood vessels in the wounds treated with CACs exposed to OPN have a significantly larger diameter than untreated wounds. Figure 5 identifies blood vessels using florescence microscopy. This technique is used to ensure blood vessels were successfully identified if there was similarity in appearance to tissue artifacts.

**Table 1:** Stereological analysis of wounds in diabetic animals

**Parameter**	**CACs (OPN) + Collagen**	**CACs + Collagen**	**Collagen**	**Untreated**
Wound volume (mm^3^)	13.2 ± 5.1*	14.1 ± 3.5	17.4 ± 3.6^#^*	12.03 ± 3.69
Total volume of inflammatory cells (mm^3^)	2.37 ± 0.97^#^	3.01 ± 1.16^#^	3.21 ± 0.91^#^	1.39 ± 0.5
Surface density of blood vessels in wound (mm^-1^)	22.4 ± 9.3^#^	21.6 ± 4.34^#^	25.6 ± 5.8^#^	11.1 ± 5
Surface area of blood vessels (mm^2^)	306.3 ± 178	330.5 ± 20^#^	442 ± 112^#^	146 ± 116
Length density of blood vessels in wound (mm^-2^)	6,212 ± 1,424^#^	6,534 ± 1,589^#^	7,575 ± 1,610^#^	4,116 ± 1093
Total length of blood vessels in wound (mm)	81,678 ± 35,117	90,490 ± 26,997^#^	131,220 ± 34,187^#^	49,337 ± 51595
Radial diffusion distance (μm)	7.29 ± 0.9^#^	7.16 ± 0.1^#^	6.56 ± 0.6^#^	9.05 ± 0.15
Vessel diameter (μm)	1.8 ± 0.47^#^	1.76 ± 0.46	1.74 ± 0.3	1.35 ± 0.38

Stereology analyzed by ANOVA followed by Fisher’s pairwise comparison, mean ± standard deviation, n = 8, *P <0.05 compared to * in the same row, ^#^P <0.05 compared to untreated wound.

Analyses of inflammatory cell infiltrate revealed a significant difference between the three treatment groups as compared to untreated wounds. Figure 6 demonstrates the use of a grid for calculating inflammatory cell infiltrate. The wounds treated with CACs exposed to OPN and delivered in a collagen scaffold revealed a trend towards reduced inflammatory cell infiltrate as compared to wounds treated with CACs and seeded in collagen and collagen alone (Table 1).

The relationship between the various healing parameters was assessed in the treatment groups using Pearson’s correlations (Table 2). For wounds treated with CACs exposed to OPN and seeded on a collagen scaffold, blood vessel diameter, length density and radial diffusion distance correlated with surface density. The increased blood vessel diameter demonstrated with this treatment correlates with surface density of blood vessels providing further evidence of a more efficient vascular network seen with this treatment. This highlights that the progression of wound healing is faster than in untreated wounds where, although there is a correlation between blood vessel diameter and surface density, it is weaker than in the CAC exposed to OPN group. Also in the untreated wound, there is a correlation between inflammatory cell infiltrate, length density and surface density. This indicates that the untreated wounds are in an earlier stage of wound healing, that is, a more inflammatory stage of wound healing. This correlation analysis indicates a more efficient vascular network in the CAC (OPN) group.

**Table 2:** Pearson’s correlation values between wound healing parameters

	**CAC (OPN) + Collagen**					**CAC + Collagen**				
	**WC**	**I**	**Sd**	**Ld**	**Rd**	**WC**	**I**	**Sd**	**Ld**	**Rd**
I	−0.02					−0.63				
Sd	−0.14	−0.19				0.53	0.01			
Ld	0.14	−0.30	0.76*			−0.55	−0.05	−0.64		
Rd	−0.15	0.30	−0.74*	−0.10		0.49	0.20	0.68	−0.98*	
BVD	−0.16	−0.02	0.86*	0.35	−0.33	−0.09	−0.44	−0.14	−0.76*	0.80*
	**Collagen**					**No treatment**				
I	−0.50					0.47				
Sd	0.09	−0.03				0.38	0.88*			
Ld	−0.20	0.35	0.70*			0.37	0.75*	0.83*		
Rd	0.16	−0.30	−0.72*	−0.99*		−0.20	−0.71*	−0.75*	−0.97*	
BVD	0.01	−0.34	0.30	−0.34	0.36	0.30	0.58	0.75*	0.27	−0.15

Numbers in bold accompanied by an * indicate statistically significant correlation coefficients (*P* <0.05). BVD, blood vessel diameter; CAC, circulating angiogenic cells seeded on a collagen scaffold; CAC (OPN), circulating angiogenic cells exposed to osteopontin and seeded on a collagen scaffold; I, inflammatory cell infiltrate; Ld, length density of blood vessels; Rd, radial diffusion distance; Sd, surface density of blood vessels; WC, percentage wound closure.

**Figure 9:**
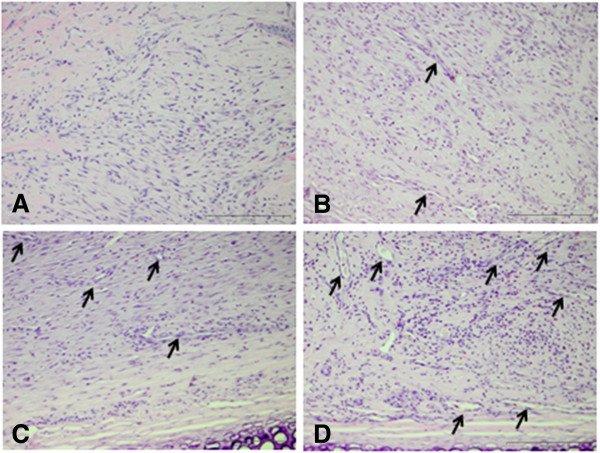
**Representative histological Images of wounds.** Hematoxylin and Eosin stained histological sections in four treatment groups are shown one week after treatment. The arrows denote blood vessels. Wounds treated with CAC (OPN) + collagen **(D)** demonstrated increased blood vessel number and diameter as compared to other groups. Collagen **(B)** and CAC + collagen **(C)** treated wounds demonstrate increased blood vessel number and length when compared to untreated wounds **(A)**. Untreated wounds have a reduced blood vessel surface area and length density as represented by the relative absence of blood vessels seen in image A when compared to other images. Scale bar = 200 μm.

Figure 9 demonstrates representative hematoxylin and eosin stained histological images of the four groups analyzed, that is, untreated wound, collagen treated wound, collagen and CAC treated wound and collagen and CAC exposed to OPN treated wound. The blood vessels are increased in number and diameter in wounds treated with collagen seeded CACs exposed to OPN.

**Figure 10:**
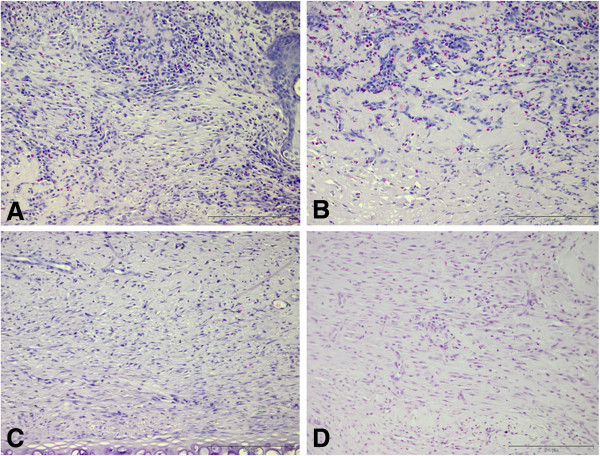
**Representative images of inflammatory cell infiltrate in wounds.** Representative images of hematoxylin and eosin stained histological sections from four treatment groups one week after treatment application are presented. The cells with dark stained nuclei represent neutrophils, macrophages and lymphocytes. Wounds treated with collagen **(A)** and CACs + collagen **(B)** reveal increased inflammatory infiltrate in comparison to control wounds **(D)**. Wounds treated with CAC (OPN) + collagen **(C)** have increased inflammatory cells when compared to control untreated wounds **(D)** with the density of inflammatory cells appearing less than wounds **(A)** and **(B)**, suggesting that wounds treated with CAC (OPN) + collagen are progressing more rapidly through the stages of wound healing in comparison to wounds treated with collagen **(A)** and CACs + collagen **(B)**. This is supported by more vascular structures evident in wounds treated with CAC (OPN) + collagen **(C)** as compared to other treatment groups. Scale bar = 200 μm. CAC, circulating angiogenic cells; OPN, osteopontin.

Figure 10 demonstrates representative hematoxylin and eosin stained histological images of the four groups analyzed. The three treatment groups (collagen treated wound, collagen and CAC treated wound and collagen and CAC exposed to OPN treated wound) demonstrate increased inflammatory cell infiltrate at one week when compared to untreated wounds. More blood vessels are evident in wounds treated with CAC (OPN) + collagen (Figure 10C).

## Discussion

Diabetic foot ulceration leads to amputation in a significant proportion of cases. There is a critical need to create novel treatments to prevent amputation in addition to decreasing time to wound closure. EPCs delivered either systemically or topically have been shown to augment wound healing in both diabetic and non-diabetic cutaneous wound models [15,16]. Early EPCs or CACs are decreased in number and are dysfunctional in people with diabetes mellitus [30]. We have shown that the exposure of CACs to OPN rescues the angiogenic potential of OPN knockout CACs and restores blood flow in limb ischemia [18]. Using this strategy to restore the disease associated dysfunction associated with diabetic CACs, the wound healing benefit of CACs exposed to OPN was tested.

Collagen was used as cells topically delivered to wounds do not remain at the wound site [31]. This research supports previous hypotheses regarding the cutaneous wound healing benefit of biomaterial mediated topical cell delivery. This new paradigm in wound healing treatments potentially increases wound healing and results in a superior neovasculature for human diabetic foot ulceration.

There is no standard animal model to study diabetic ulceration. The impaired cutaneous wound healing associated with diabetes mellitus in humans may be due to a large number of factors [6]. It is not possible to fully replicate these factors in animal models. The hyperglycemic rabbit ear ulcer is similar to the human diabetic foot ulcer as it heals by epithelialization and granulation tissue formation. The rabbit ear ulcer wound heals from the wound edge. This is an advantage of the model as the cutaneous wounds heal with inflammatory cells infiltrating the wound edge in addition to angiogenesis and granulation tissue formation. This allows for assessment of these wound healing phases at the area of the wound where new wound healing is occurring. The use of stereology allows determination of inflammatory cell infiltrate and angiogenesis at this area of wound healing. As several wounds can be created on each ear, the effects of different treatments can be assessed and compared. The rabbit ear ulcer does not heal by skin contraction. This is advantageous over cutaneous wounds in rodents which heal by contraction. Apart from wound contraction, the wound healing in rodents occurs by similar mechanisms as in the diabetic rabbit ear ulcer. In rodents, the measurement of percentage wound closure is difficult, as a reduction in the size of the wound area is due to contraction of the skin and not entirely due to inflammation, neoangiogenesis and granulation tissue formation as is the normal paradigm in adult human cutaneous wound healing. There are advantages to the use of mice or rat models. These models are more widely studied. There are more antibodies available for rodent models and these animals are less expensive.

A unique aspect of the diabetic rabbit ear ulcer model of cutaneous ulceration is that the wound is in close proximity to the underlying cartilage. This is not identical to the human wound healing situation but does provide a valid means for analyzing wound healing. There is minimal adipose tissue underlying the wound in this model. The significance of this is not clear, as the role of adipose tissue in wound healing is not fully elucidated. Adipose-derived stem cells promote wound healing and enhance the functions of keratinocytes and dermal fibroblasts [32]. The reduced adipose tissue layer may influence wound healing but this does not invalidate the model. In humans, diabetic neuropathic ulceration occurs on the plantar aspect of the foot at areas of increased pressure which has minimal adipose tissue, highlighting a further similarity to diabetic wound healing in humans.

After five weeks of hyperglycemia, wounds were created. One week after wound creation, percentage wound closure was less in the diabetic animals as compared to non-diabetic animals. These data validate that the animal model represents a model of compromised wound healing. Further endpoints including inflammatory cell infiltrate and angiogenesis were not assessed in diabetic and non-diabetic wounds. It is known that a chronic inflammatory wound microenvironment persists in the diabetic ulcer and that non-healing ulceration is associated with an impaired vascular supply [7]. A change in the inflammatory response in cutaneous wounds may alter the rate of wound healing and the difference in percentage wound closure may be attributable to a difference in inflammatory responses in diabetic and non-diabetic wounds. However, the analysis of inflammatory cell infiltrate in diabetic and non-diabetic wounds would not affect the testing of the main hypothesis in this study. The endpoint of percentage wound closure in diabetic wounds at one week is significantly less than in non-diabetic wounds, and the preclinical model of impaired wound healing was deemed suitably robust to assess the wound healing effect of topically applied autologous CACs exposed to OPN and seeded on a collagen scaffold.

Metabolic activity is the assessment of mitochondrial activity in a cell. It reflects cellular health and cellular viability. It refers to oxidation-reduction reactions of the electron transport chain in mitochondria. Increased metabolic activity signifies increased cellular health and viability. AlamarBlue® monitors the reducing environment of the living cell by measuring these oxidation-reduction reactions. An increase in metabolic activity indicates an increase in the activity of the electron transport chain in mitochondria and therefore, an increase in metabolic activity in a cell [33,34]. Rabbit CACs seeded on a collagen scaffold demonstrated increased metabolic activity pre-transplantation. This increased metabolic activity is in comparison to control scaffold which did not demonstrate any increased metabolic activity. This result demonstrates that the isolation and seeding of the collagen scaffold protocols used are effective and result in viable cells being transplanted to a wound.

Florescently labeled rabbit CACs seeded in a collagen scaffold were identified in the wound one week after treatment. The metabolic activity of 5 × 10^4^ CACs reduced to control levels by 72 hours *in vitro*. The detection of cells in the wound after one week ensures the collagen effectively mediates cell delivery to the wound.

Topical CAC therapy was investigated on wounds created after five weeks of hyperglycemia. Autologous CACs were successfully isolated from peripheral blood of hyperglycemic rabbits and cells were exposed to OPN *ex vivo*. These cells were then topically re-administered to a full thickness cutaneous ulcer via a collagen scaffold. The treatment group with CACs exposed to OPN and delivered using a collagen scaffold demonstrated increased percentage wound closure at one week post treatment as compared to collagen alone and untreated wounds. Percentage wound closure is a clinically relevant endpoint in wound healing research and a wound that closes more quickly is a goal for assessment of wound healing treatment efficacy. This was not observed in wounds treated with collagen and CACs not exposed to OPN. This highlights the crucial role of pretreatment of cells with the matricellular protein OPN in order to achieve an increased wound healing response.

Stereology is a robust scientific tool for determining angiogenesis and tissue responses to tissue engineered constructs *in vivo*. An extensive analysis was performed through one half of each wound at 150 micrometer intervals. The beneficial increase in percentage wound closure is not fully explained by an increase in the angiogenesis endpoints of length density and surface density of blood vessels. A significantly increased surface density and length density, in addition to a reduced radial diffusion distance, is noted with all treatment groups in comparison to untreated wounds (Table 1). It can be concluded that angiogenesis is supported in the three treatment groups. This is in keeping with previous observations that type 1 collagen is known to support angiogenesis [35]. As the current methodology is an extensive analysis through the wound this is regarded as a representative sample from wounds. Blood vessels in the wounds treated with CACs exposed to OPN have a significantly larger diameter than untreated wounds suggesting a more accelerated wound healing response with more mature blood vessels.

Analyses carried out for inflammatory cell infiltrate revealed a significant difference between the three treatment groups as compared to untreated wounds. The wounds treated with CACs exposed to OPN and delivered in a collagen scaffold revealed a trend to a reduced inflammatory cell infiltrate as compared to wounds treated with CACs and seeded in collagen and collagen alone (Figure 10). In addition, there are more vascular structures evident in wounds treated with CACs exposed to OPN and delivered in a collagen scaffold (Figure 9D). This result highlights a potentially reduced inflammatory environment and more mature vasculature in wounds treated with CACs exposed to OPN. This response is beneficial as non-healing diabetic ulcers are associated with an increased inflammatory cell environment [7].

The initial stages of wound healing are characterized predominantly by inflammation and immature blood vessels. As normal adult wound healing progresses through successive phases, the wound tissue comprises of a reduced inflammatory environment and a more mature vasculature. It can be hypothesized that treatment of wounds with CACs exposed to OPN and seeded on a collagen scaffold accelerates the normal wound healing response and may release the diabetic ulcer from an abnormal wound healing paradigm to a more physiological phenotype.

## Conclusions

In conclusion, an animal model of impaired wound healing in diabetes is described. The hyperglycemic rabbit ear ulcer model investigated is a valid preclinical model for assessing the effect of topical therapies using the end point of wound closure. This is a model which more closely resembles the human condition in comparison to other rodent models. Extensive histological analysis provides insight into the neovasculature in healing wounds. This investigative approach is relevant as a central pathological process in non-healing diabetic ulceration is impaired vascular supply. Subsequently, a scaffold based cell transfer system is demonstrated. The cell scaffold treatment approach allows for *ex vivo* manipulation of autologous cells in addition to successfully mediating cell delivery to a wound. The treatment demonstrates retention of metabolic activity of cells and positioning of cells at the wound site. Finally, this treatment results in augmented healing with OPN-modified CACs seeded on collagen in comparison to other groups. It is shown that all collagen groups had increased angiogenesis. However, treatment of collagen seeded with CACs exposed to OPN results in a more efficient neovasculature and evidence of more fully healed wounds.

## Abbreviations

ANOVA: analysis of variance; CAC: circulating angiogenic cell; DiI-Ac-LDL: DiI-acetylated-low-density lipoprotein; EDTA: ethylenediaminetetraacetic; EPC: endothelial progenitor cell; H & E: hematoxylin and eosin; OPN: osteopontin; PBS: phosphate-buffered saline.

## Competing interests

Timothy O’Brien is a founder, director and equity holder in Orbsen Therapeutics Ltd. For all other authors there is no conflict of interest.

## Authors’ contributions

TOB, AP and AOL were responsible for the conception and design of the study AOL, MK and MC were responsible for performing *in vitro* experiments, animal experiments and stereology. AOL, MK, EEV, AOL, PD, AP and TOB were responsible for analyzing the data. AOL, TOB, AP, MK and AL were responsible for drafting the manuscript. All authors read and improved the final manuscript.
